# Depressive Symptoms in Adolescence and Young Adulthood

**DOI:** 10.1001/jamanetworkopen.2024.27748

**Published:** 2024-08-14

**Authors:** Katherine M. Keyes, Noah T. Kreski, Megan E. Patrick

**Affiliations:** 1 Department of Epidemiology, Mailman School of Public Health, Columbia University, New York, New York; 2 Institute for Social Research, University of Michigan, Ann Arbor

## Abstract

**Importance:**

Depressive symptoms have increased among US adolescents since 2010. It remains unclear as to what extent this increase will persist into young adulthood, potentially turning the youth mental health crisis into a young adult mental health crisis.

**Objective:**

To test the association between birth cohort and adolescent depressive symptoms at ages 18, 19 to 20, and 21 to 22 years and changes in these symptoms by cohort.

**Design, Setting, and Participants:**

This panel cohort study analyzed data from the Monitoring the Future longitudinal survey from 1990 to 2019, including birth cohorts from 1972 to 2001. Survey respondents were recruited from US high schools in 12th grade and were approximately aged 18 years (at baseline) through age 21 to 22 years (during mail and web follow-up). Data analysis was conducted from April to October 2023.

**Exposure:**

Depressive symptoms score (>12 vs ≤12, with >12 representing top-decile scores) on a scale examining affective items (eg, “Life often seems meaningless”).

**Main Outcomes and Measures:**

High (vs lower) depressive symptoms at ages 19 to 20 years and 21 to 22 years.

**Results:**

The 36 552 respondents included 18 597 females (50.5%), and most reported having a parent who graduated from college (44.8%). Among females, 19.1% (95% CI, 16.7%-21.4%) of the most recent birth cohort (born: 1997-2001) had high depressive symptoms at age 18 years, higher than any previous birth cohort. While prevalence declined by age 21 to 22 years, it remained higher than previous cohorts at that age. Among males, 13.4% (95% CI, 11.2%-15.6%) of the most recent birth cohort had high depressive symptoms at age 18 years, and prevalence increased through young adulthood. Males with high baseline depressive symptoms had 10.24 (95% CI, 7.01-14.97) times the odds of symptoms at age 19 to 20 years and 6.20 (95% CI, 3.93-9.78) times the odds of symptoms at age 21 to 22 years. Females with high baseline depressive symptoms had 9.16 (95% CI, 6.57-12.76) times the odds of symptoms at age 19 to 20 years and 7.28 (95% CI, 4.92-10.78) times the odds of symptoms at age 21 to 22 years. The magnitude of the associations did not vary over time. Population attributable fractions indicated that the total proportion of young adult symptoms associated with depressive symptoms at age 18 years among females has increased; in the most recent birth cohort, 55.25% (95% CI, 38.11%-65.13%) of depressive symptoms at age 21 to 22 years were associated with symptoms at age 18 years.

**Conclusions and Relevance:**

This panel cohort study found that increases in depressive symptoms in adolescence persisted into young adulthood, suggesting the need for primary prevention and mental health resources during the adolescent years.

## Key Points

**Question:**

To what extent have increases in adolescent depressive symptoms in recent birth cohorts persisted among young adults?

**Findings:**

In this cohort study of 36 552 survey respondents, depressive symptoms at age 18 years were associated with depressive symptoms at ages 19 to 20 and 21 to 22 years, and this association did not vary by birth cohort. Increasing depressive symptoms in adolescence persisted into young adulthood, and a larger proportion of young adult symptoms in females was associated with symptoms during adolescence.

**Meaning:**

Findings of this study suggest the need for primary prevention and mental health resources during the adolescent years.

## Teaser

This cohort study examines the persistence of depressive symptoms and other mental health issues from age 18 years to age 21 to 22 years.

## Introduction

Depressive symptoms have been increasing among US adolescents since approximately 2010. National estimates of major depressive episode prevalence among adolescents increased from 8.1% in 2009 to 15.9% in 2019, with increases concentrated among females and ranging from 11.4% to 23.4%.^1^ Approximately 1 in 4 adolescent females reported seriously considering suicide and making a plan for suicide in 2021,^2^ an increase from approximately 14% and 11%, respectively, in 2009.^3^ Hospitalizations for intentional self-harm^4^ and fatal suicide^5,6^ also increased during the same time frame. While fatal suicide generally plateaued during the COVID-19 pandemic,^7^ symptoms of mental health problems have continued to escalate.^2,8,9,10,11^ Together, these patterns have been labeled as a teen mental health crisis in the US.

An open question is the extent to which increases in adolescent depressive symptoms in recent birth cohorts are associated with subsequent increases in young adult mental health problems as those cohorts transition into adulthood. In a given person, depressive symptoms at 1 developmental time point are typically associated with depressive symptoms at subsequent time points^12,13^; thus, the population-wide increase in the prevalence of adolescent depressive symptoms will, based on historical data, affect later developmental time points, increasing the overall population burden in young adulthood. Yet the rapid increases in depressive symptoms in the past decade have been unprecedented; thus, whether these symptoms tend to resolve more frequently than in previous birth cohorts is unknown.

A relevant epidemiological question can be framed around the interaction between birth cohort and adolescent depressive symptoms. If adolescent depressive symptoms are developmentally limited (ie, more likely to resolve before adulthood than in previous birth cohorts), then we could expect an inverse interaction between birth cohort and these symptoms. That is, if there is an inverse interaction, then the association between adolescent and young adult depressive symptoms would have lower magnitude in recent birth cohorts than in previous birth cohorts. If there is no interaction between birth cohort and adolescent depressive symptoms, then these symptoms would be as much of a factor in young adult symptoms in recent birth cohorts as in previous birth cohorts. In the context of the rising prevalence of adolescent depressive symptoms, no interaction would portend a greater burden of young adult symptoms in recent years. Another potential scenario is a direct interaction, whereby the association between adolescent and young adult depressive symptoms increases in magnitude in recent birth cohorts. A direct interaction would imply that an increased prevalence of adolescent depressive symptoms makes these symptoms more persistent into young adulthood in recent than in previous birth cohorts, suggesting a higher mental health burden in this population and warranting stronger interventions.

In the present study, we used nationally representative longitudinal data collected from adolescence through young adulthood across cohorts spanning the past 30 years to test the association between birth cohort and adolescent depressive symptoms at ages 18, 19 to 20, and 21 to 22 years and changes in these symptoms by cohort. We examined the extent to which increases in depressive symptoms and other measures of mental well-being in adolescence persist into young adulthood and whether this persistence changes across birth cohorts given the dynamic temporal patterns in recent years.

## Methods

Data for analysis were drawn from the Monitoring the Future (MTF) study, a longitudinal panel cohort study wherein a new prospective cohort began to be followed every year starting at age 18 years, selected from a nationally representative sample of high school seniors. The University of Michigan Institutional Review Board (IRB) approved the study, and the Columbia University IRB approved the analysis protocols. Passive (letter sent to parents/caregivers) or active (written) informed consent was obtained, per school policy, at baseline from parents of participants younger than 18 years or from participants 18 years or older. We followed the Strengthening the Reporting of Observational Studies in Epidemiology (STROBE) reporting guideline.

The pool of eligible respondents included those who participated in nationally representative samples of approximately 15 000 US high school seniors (12th grade), and we included data from those surveyed annually since 1990.^14^ Among that pool, approximately 2450 students were randomly selected each year for longitudinal follow-up, with oversampling for students who reported drug use (statistical weights in analysis adjusted for oversampling).^15^ Students selected for follow-up were randomly assigned to begin assessments either 1 year after baseline (modal age, 19 years) or 2 years after baseline (modal age, 20 years) and were then followed up every 2 years (either at 19 to 21 years or 20 to 22 years for the first 2 follow-up waves). In the 12th grade, respondents provided their name and contact information. This contact information was used for mailing invitation letters and sending emails and text messages. The MTF panel surveys were historically mailed paper surveys; however, starting in 2018, web-based surveys were introduced, with a paper option for those who preferred it. Participants were randomized to receive some subsets of measures, including mental health modules.

The present study included survey respondents who were queried about depressive symptoms and other measures of well-being. We included panel data for cohorts who were in the 12th grade from 1990 to 2019 (birth cohorts: 1972-2001) and followed up at ages 19 to 20 years (follow-up 1) and 21 to 22 years (follow-up 2) from 1991 to 2020 (with 36 552 participants queried about mental health). This population reflects approximately half of the total sample across birth cohorts.

Attrition from the study cohort increased across age and has increased across cohorts. The most important source of attrition was between baseline and first follow-up. Among the subset included in this analysis, the attrition range across study years was from 25.8% for those in the 12th grade in 1991 to 69.1% for those in the 12th grade in 2015. Similarly, by age 21 to 22 years, attrition for cohorts ranged from 33.4% for those in the 12th grade in 1991 to 68.7% for those in the 12th grade in 2015. We stratified by 5-year cohorts. Sample sizes by birth cohort and follow-up wave are provided in eTable 1 in Supplement 1. We included attrition weighting in all analyses that calculated individual weights as the inverse of the probability of participation based on time-invariant characteristics measured at 12th grade.^15,16^ Previous simulation analyses indicated that, while attrition varies by cohort, there is minimal resulting bias.^17^

### Measures

We examined 4 separate mental health scales: depressive affect, self-derogation, low self-esteem, and loneliness. Four items each were used to measure depressive affect, self-derogation, and low self-esteem, while 3 items were used to measure loneliness based on the extent to which respondents agreed or disagreed with certain statements. Example statements were as follows: “Life often seems meaningless” (depressive affect), “I take a positive attitude toward myself” (low self-esteem when reverse-coded), “I feel I do not have much to be proud of” (self-derogation), and “A lot of times I feel lonely” (loneliness). Response options for each item ranged from 1 (disagree) to 5 (agree). Items for each scale were summed to yield scores ranging from 4 to 20 (or from 3 to 15 for the loneliness scale), with higher scores indicating higher depressive affect, self-derogation, and loneliness, whereas a lower score on the self-esteem scale indicated worse self-esteem. Scale items were similar to existing scales with high reliability.^18,19,20^ These scales have been operationalized and implemented similarly in other analyses of MTF data.^21,22^

Due to symptom score non-normality, we created binary indicators. The main factor was depressive symptoms score at age 18 years (>12 vs ≤12, with >12 representing the top decile across all years or cohorts). Additional factors included self-derogation symptoms (>14 vs ≤14), self-esteem (≤11 vs >11), and loneliness symptoms (>13 vs ≤13) scores at age 18 years, with cutoff scores based on the top decile across all years or cohorts. Outcomes included the same mental health scale dichotomizations at ages 19 to 20 and 21 to 22 years.

### Covariates

Due to potential confounding, participants were queried about their sex (male vs female), race and ethnicity, and parental educational level at baseline (at least 1 parent was a college graduate vs no college). Until 2005, respondents selected 1 racial and ethnic category, including American Indian (Native American Indian), Asian American, Black or African American, Cuban American, Mexican American or Chicano, Other Latin American, Puerto Rican, White or Caucasian, or other. From 2005 onward, respondents could select multiple categories from the following: American Indian or Alaska Native, Asian American, Black or African American, Cuban American, Mexican American or Chicano, Native Hawaiian or Other Pacific Islander, Other Hispanic or Latino, Puerto Rican, or White. Across years, we harmonized the categories as follows: American Indian or Alaska Native, Asian or Pacific Islander, Black, Hispanic or Latino, non-Hispanic multiracial (based on reporting multiple racial identities), and White. For respondents surveyed before 2005, we also categorized as other those who selected that option. To account for potential response differences due to the COVID-19 pandemic, we included a dichotomous variable for data collection in 2020 vs all other years.

### Statistical Analysis

Analyses included descriptive graphs and logistic regression models conducted from April to October 2023 using survey procedures in SAS 9.4 (SAS Institute Inc). Descriptive prevalence estimates included sampling weights. Logistic regression models estimated the association between mental health at age 18 years and mental health at ages 19 to 20 and 21 to 22 years, incorporating attrition weights and controlling for race and ethnicity, parental educational level, birth cohort, and an indicator for data collection in 2020 vs previously. Interactions with birth cohort and sex were also tested. Two-sided *P* < .05 indicated statistical significance.

Additionally, we estimated the population attributable fraction (PAF), which measures the total proportion of outcomes that can be attributed to the exposure and is calculated as follows:

*PAF* = *p*(*RR* − 1)/[*p*(*RR* − 1) + 1],

where *p* is the exposure prevalence, and *RR* (relative risk) is the relationship between the exposure and outcome.^23^ In this study, the exposure was depressive symptoms at age 18 years, and the outcomes were depressive symptoms at ages 19 to 20 and 21 to 22 years.

Population attributable fractions are community impact measures that change based on both the prevalence of exposure or independent variables and the magnitude of the association between exposure or independent variables and outcomes. The PAF increases as exposure prevalence increases, holding RR constant, and increases as RR increases, holding prevalence constant.

## Results

### Prevalence of High Depressive Symptoms and Change in Depressive Symptoms

Of the 36 552 respondents who were followed-up longitudinally, 18 597 (50.5%) were females and 17 687 (48.8%) were males; 1.1% identified as American Indian or Alaska Native, 3.3% as Asian or Pacific Islander, 10.5% as Black, 14.0% as Hispanic or Latino, 66.0% as White, 1.8% as non-Hispanic multiracial, and 1.7% as other race and ethnicity; and 44.8% had a parent who graduated from college (eTable 2 in Supplement 1).

**Figure 1.**
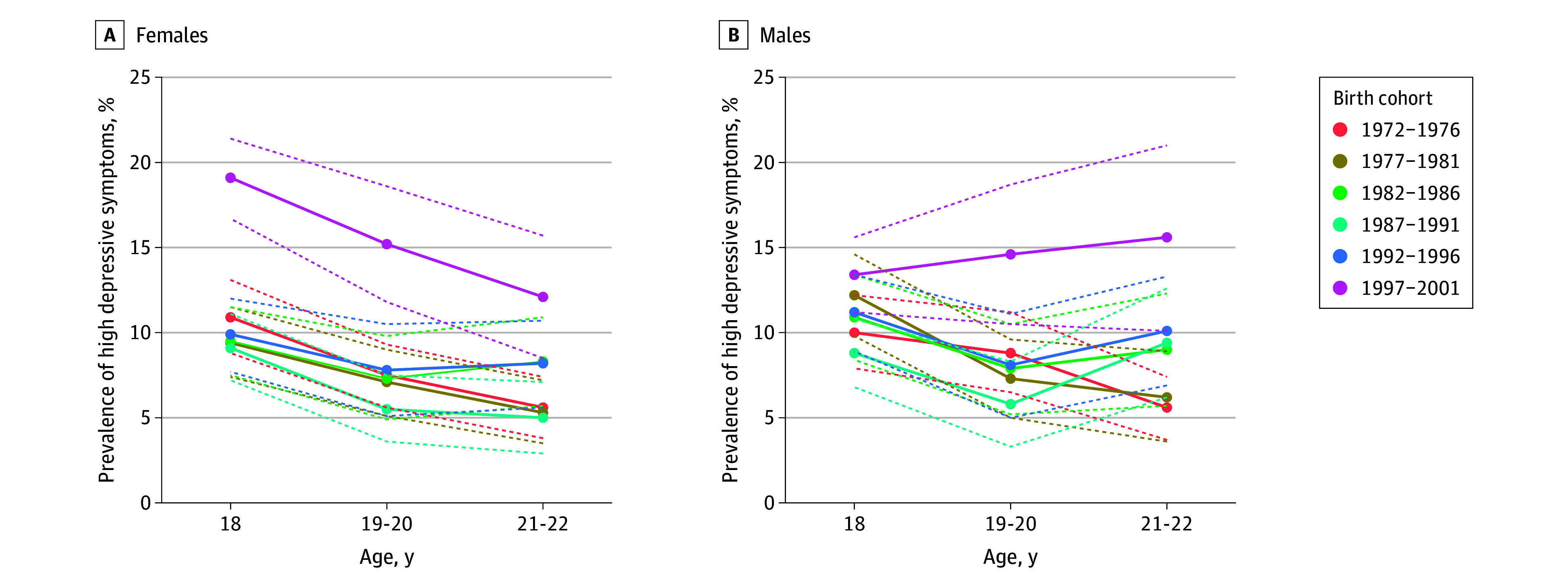
Longitudinal Prevalence of High Depressive Symptoms by Birth Cohort and Sex Among US Young Adults Born From 1972 Through 2001 and Measured From 1990 Through 2019

Among females (Figure 1), 19.1% (95% CI, 16.7%-21.4%) of those in the most recent birth cohort (born 1997-2001) had high depressive symptoms at age 18 years, which was higher than any other previous birth cohort; while depressive symptom prevalence decreased to 12.1% (95% CI, 8.5%-15.7%) by age 21 to 22 years, it remained higher than any previous birth cohort at age 21 to 22 years. Among males (Figure 1), 13.4% (95% CI, 11.2%-15.6%) of those in the most recent birth cohort (born 1997-2001) had high depressive symptoms at age 18 years, which was higher than any other previous birth cohort, and symptom prevalence increased to 15.6% (95% CI, 10.1%-21.0%) by age 21 to 22 years. Birth cohort patterns for loneliness, self-derogation, and low self-esteem are provided in eFigures 1, 2, and 3 in Supplement 1.

Compared with the cohort born from 1972 to 1976, the cohort born from 1997 to 2001 had elevated depressive symptoms at age 18 years (adjusted odds ratio [AOR], 1.70; 95% CI, 1.38-2.11), 19 to 20 years (AOR, 1.91; 95% CI, 1.37-2.66), and 21 to 22 years (AOR, 2.35; 95% CI, 1.46-3.78) (Table 1). Increases among recent birth cohorts were also evident across other domains of mental health and well-being (eTable 3 in Supplement 1), including self-derogation, low self-esteem, and (to a lesser extent) loneliness.

**Table 1.** Adjusted Odds Ratios (AORs) Between Birth Cohort and High Depressive Symptoms at Ages 18, 19 to 20, and 21 to 22 Years^a^

Birth cohort	Depressive symptoms at age 18 y		Depressive symptoms at age 19-20 y		Depressive symptoms at age 21-22 y	
	AOR (95% CI)	No.	AOR (95% CI)	No.	AOR (95% CI)	No.
Total		12 465		7251		6460
**Overall**						
1972-1976	1 [Reference]	1951	1 [Reference]	1496	1 [Reference]	1382
1977-1981	1.04 (0.82-1.33)	1911	0.93 (0.68-1.28)	1354	1.06 (0.72-1.56)	1198
1982-1986	0.96 (0.74-1.24)	1876	0.82 (0.57-1.16)	1208	1.51 (1.03-2.23)^b^	1097
1987-1991	0.88 (0.68-1.13)	1888	0.68 (0.46-0.99)^b^	1024	1.27 (0.85-1.89)	975
1992-1996	1.01 (0.79-1.30)	1923	0.97 (0.67-1.40)	960	1.75 (1.21-2.54)^b^	1165
1997-2001	1.70 (1.38-2.11)^b^	2916	1.91 (1.37-2.66)^b^	1209	2.35 (1.46-3.78)^b^	643
**Males**						
No.		5866		3049		2705
1972-1976	1 [Reference]	955	1 [Reference]	683	1 [Reference]	603
1977-1981	1.34 (0.94-1.89)	928	0.87 (0.55-1.40)	578	0.95 (0.53-1.68)	507
1982-1986	1.19 (0.82-1.73)	865	0.83 (0.49-1.40)	477	1.49 (0.84-2.65)	431
1987-1991	0.93 (0.64-1.35)	887	0.73 (0.42-1.29)	417	1.54 (0.89-2.69)	408
1992-1996	1.16 (0.82-1.66)	929	0.85 (0.49-1.49)	414	1.87 (1.10-3.16)^b^	493
1997-2001	1.52 (1.10-2.11)^b^	1302	1.92 (1.16-3.17)^b^	480	2.77 (1.37-5.59)^b^	263
**Females**						
No.		6497		4172		3744
1972-1976	1 [Reference]	996	1 [Reference]	813	1 [Reference]	779
1977-1981	0.83 (0.58-1.17)	983	0.99 (0.65-1.51)	776	1.11 (0.66-1.85)	691
1982-1986	0.79 (0.56-1.12)	1011	0.79 (0.49-1.29)	731	1.46 (0.86-2.49)	666
1987-1991	0.84 (0.60-1.19)	1001	0.64 (0.38-1.08)	607	0.96 (0.53-1.72)	567
1992-1996	0.90 (0.64-1.28)	994	1.11 (0.68-1.81)	546	1.61 (0.96-2.70)	672
1997-2001	1.92 (1.44-2.56)^b^	1512	1.90 (1.24-2.92)^b^	699	1.82 (0.99-3.35)	369

^a^ Adjusted for race and ethnicity, parental educational level, and pre- or post-2020 indicator.

^b^ *P* < .05.

### Longitudinal Associations and Interactions 

Table 2 shows the overall longitudinal associations of depressive symptoms across follow-up waves. Among males, those with high depressive symptoms at baseline had 10.24 (95% CI, 7.01-14.97) times the odds of high depressive symptoms at age 19 to 20 years and had 6.20 (95% CI, 3.93-9.78) times the odds of high depressive symptoms at age 21 to 22 years. Among females, those with high depressive symptoms at baseline had 9.16 (95% CI, 6.57-12.76) times the odds of high depressive symptoms at age 19 to 20 years and 7.28 (95% CI, 4.92-10.78) times the odds of high depressive symptoms at age 21 to 22 years. eTable 4 in Supplement 1 shows similarly poor mental health outcomes across follow-up waves as indicated by baseline depressive symptoms as well as baseline self-derogation, loneliness, and low self-esteem.

**Table 2.** Adjusted Odds Ratios (AORs) Between Baseline Mental Health Factors and Mental Health Outcomes at Ages 19 to 20 and 21 to 22 Years^a^

Depressive symptoms by birth cohort	Depressive symptoms at age 18 y, AOR (95% CI)	*P* value^b^
Overall		
Age 19-20 y	9.57 (7.45-12.28)	.61
Age 21-22 y	6.37 (4.75-8.55)	.70
Males		
Age 19-20 y	10.24 (7.01-14.97)	.39
Age 21-22 y	6.20 (3.93-9.78)	.60
Females		
Age 19-20 y	9.16 (6.57-12.76)	.79
Age 21-22 y	7.28 (4.92-10.78)	.19

^a^ Adjusted for race and ethnicity, parental educational level, cohort, and pre- or post-2020 indicator.

^b^ Interaction between depressive symptoms by cohort and at age 18 y.

A critical question in this investigation was whether there were interactions between birth cohort and depressive symptoms at age 18 years; that is, whether the association between depressive symptoms at age 18 years and depressive symptoms at both age 19 to 20 years and age 21 to 22 years was changing across birth cohort. Table 2 shows no interaction among both females and males. There was no evidence of meaningful interactions between birth cohort and depressive symptoms at age 18 years compared with depressive symptoms at age 19 to 20 years or age 21 to 22 years.

### Depressive Symptom Prevalence and PAFs 

**Figure 2.**
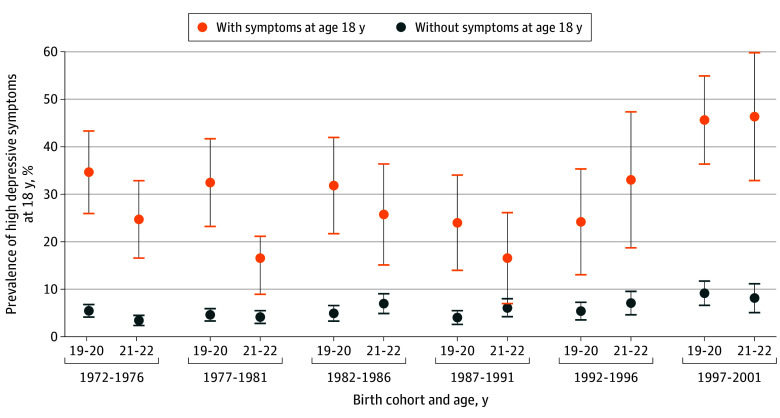
Prevalence of High Depressive Symptoms at Ages 19 to 20 and 21 to 22 Years, by Birth Cohort and Presence of High Depressive Symptoms at Age 18 Years Error bars represent 95% CIs.

Figure 2 shows follow-up depressive symptoms stratified by whether adolescents had high depressive symptoms at baseline. Unadjusted regression models did not find significant interactions between baseline depressive symptoms and sex (eTable 5 in Supplement 1); thus, Figure 2 grouped males and females together. Results indicated that the presence of high depressive symptoms at each follow-up were concentrated among those with high depressive symptoms at age 18 years. By the most recent birth cohort, among those with high depressive symptoms at baseline, approximately 45.6% had high depressive symptoms at age 19 to 20 years and 46.3% had high depressive symptoms at age 21 to 22 years. Comparatively, in previous birth cohorts, the prevalence of high depressive symptoms at follow-up waves among those with high depressive symptoms at baseline ranged from 16.5% to 34.6%. Analyses for loneliness, self-derogation, and low self-esteem are available in eFigures 4, 5, and 6 in Supplement 1.

Table 3 shows the PAFs for the proportion of high depressive symptoms at ages 19 to 20 and 21 to 22 years attributable to high depressive symptoms at age 18 years. The results indicated that among these birth cohorts, the proportions of high depressive symptoms at ages 19 to 20 and 21 to 22 years were highly associated with depressive symptoms at age 18 years. In earlier cohorts, PAFs were typically larger for males; for later cohorts, PAFs were typically larger for females. While there is no formal statistical test for trend in PAFs, we found that among females born from 1972 to 1976, 35.28% (95% CI, 19.42%-50.14%) of depressive symptoms at age 21 to 22 years were associated with depressive symptoms at age 18 years, whereas among females born from 1997 to 2001, 55.25% (95% CI, 38.11%-65.13%) of depressive symptoms at age 21 to 22 years were associated with depressive symptoms at age 18 years. Overall, in the most recent cohort born from 1997 to 2001, 39.38% (95% CI, 30.74%-46.37%) of high depressive symptoms at age 19 to 20 years were associated with depressive symptoms at age 18 years, and 43.44% (95% CI, 30.98%-52.44%) of high depressive symptoms at age 21 to 22 years were associated with depressive symptoms at age 18 years.

**Table 3.** Population Attributable Fractions (PAFs) for the Proportion of High Depressive Symptoms in Young Adults Associated With High Depressive Symptoms at Age 18 Years

Birth cohort	PAF (95% CI)	
	High depressive symptoms at age 19-20 y	High depressive symptoms at age 21-22 y
**Overall**		
1972-1976	35.99 (27.38-43.68)	39.51 (27.83-50.10)
1977-1981	39.48 (29.30-48.34)	24.45 (12.05-37.85)
1982-1986	35.86 (24.64-45.79)	21.56 (11.05-32.43)
1987-1991	30.79 (18.13-43.13)	13.34 (3.34-26.03)
1992-1996	26.81 (13.77-39.81)	27.78 (14.92-39.44)
1997-2001	39.38 (30.74-46.37)	43.44 (30.98-52.44)
**Males**		
1972-1976	37.29 (24.92-47.33)	43.52 (26.72-57.06)
1977-1981	43.31 (26.79-56.43)	25.69 (7.07-45.89)
1982-1986	43.30 (26.00-56.52)	21.93 (5.91-38.39)
1987-1991	30.68 (12.53-47.66)	11.35 (0.32-25.71)
1992-1996	28.53 (9.09-47.40)	27.71 (9.28-43.56)
1997-2001	33.68 (20.01-44.16)	30.26 (12.13-43.78)
**Females**		
1972-1976	34.64 (22.74-45.26)	35.28 (19.42-50.14)
1977-1981	35.75 (23.26-46.44)	22.90 (8.13-39.83)
1982-1986	29.73 (15.60-42.72)	21.04 (8.06-34.65)
1987-1991	30.61 (14.24-46.78)	17.11 (0.78-39.84)
1992-1996	25.34 (9.11-41.74)	27.92 (11.04-43.05)
1997-2001	43.61 (31.83-52.19)	55.25 (38.11-65.13)

## Discussion

Depressive symptoms and other indicators of mental health and well-being are increasing in US adolescents and young adults. The magnitude of the association between having depressive symptoms at age 18 years and having depressive symptoms at ages 19 to 20 and 21 to 22 years has remained stable across birth cohorts, yet prevalence across all ages is increasing by cohort. Thus, the increases in youth mental health problems in the US are shifting to young adults as populations age. By the most recent birth cohort, nearly half of high depressive symptoms at age 21 to 22 years were associated with high symptoms in adolescence; thus, early intervention and prevention are critical to reduce the burden on both adolescent and adult populations.

Previous studies examining the development of depressive symptoms from adolescence to young adulthood have shown that, in a given individual, symptom prevalence is associated with time points yet is often heterogeneous across the lifespan.^9,10,11^ Ongoing studies often follow cohorts of similar age at baseline across time, and thus a broader historical lens is often not possible with existing cohorts. Data from other national longitudinal sources, such as the National Longitudinal Study of Adolescent to Adult Health, have shown that depressive symptoms typically decline after adolescence and into young adulthood^24^ and then accelerate, particularly for females, at the start of midlife.^24^ Data from the present study were generally consistent with this pattern, especially for females, revealing decreases in depressive symptoms after age 18 years. However, this study adds to the literature by showing a substantial cohort variation in depressive symptom trajectory, with mean levels of depressive symptoms increasing in the most recent birth cohorts, across the age span of adolescence to young adulthood.

The PAFs indicate that, across all cohorts, adolescent depressive symptoms explain a substantial proportion of young adult depressive symptoms and that the proportions explained appear to be increasing for females. The PAF is an important component of evaluating the implications of increasing depressive symptoms by cohort^25,26^ because it has 2 central components: magnitude of associations and prevalence. It increases as prevalence increases, even when the relationship between exposure and outcome remains steady. Given that we did not observe an interaction between birth cohort and depressive symptoms at age 18 years, the reason that the PAF increased for females was that prevalence at age 18 years was increasing. Thus, without considering PAFs, we might erroneously conclude that the public health burden of these symptoms is remaining stable for females since the magnitude of association is not changing. But the public health burden is increasing for females because the exposure prevalence is increasing, even while the magnitude of the relationship is stable across cohorts. In other words, in the case of adolescent to adult depressive symptoms across birth cohorts, the public health burden for females is increasing due to the increase in depressive symptoms at age 18 years.

Increases in adolescent, and now young adult, mental health symptoms indicate a growing need for additional infrastructure for treatment and services. Available data indicate that there are substantial gaps in availability of trained specialist mental health practitioners across the US^27^ as well as access challenges even in areas in which practitioners are concentrated. Psychiatrists are less likely to accept insurance than other clinicians,^28^ creating financial barriers for most US families. Financial barriers are among the most frequently cited reasons for unmet mental health care needs among people with psychiatric distress.^29^ Medicaid remains an important payer of mental health services in the US for individuals eligible for coverage, but from 2010 to 2015—a period of the Affordable Care Act implementation and Medicaid expansion in many states—the proportion of psychiatrists who accepted Medicaid decreased.^30^ Primary care physicians and pediatricians are increasingly a central source of mental health care for US adolescents and adults,^31^ but the lack of specialized training for more complex mental health challenges may continue the treatment gaps. Rates of emergency department use for mental health care among Medicaid recipients differ up to 8-fold across US regions,^32^ suggesting access gaps. While expansion of teletherapy and telehealth, as well as the launch of the 3-digit mental health crisis line, may be leveraged to expand mental health care access, it is clear that primary prevention, ideally before upcoming cohorts reach adulthood, is also urgently needed. Beyond the expansion of the psychiatric workforce, school- and family-based interventions for adolescents to learn evidence-based stress-coping strategies are also critical to improving population mental health.^33^

The underlying factors associated with increases in depressive symptoms among adolescents remain under investigation. Substantial resources have been placed in identifying the role of digital technology and social media in accelerating mental health problems among youth, although thus far meta-analyses and systematic reviews have generally found small to null effect sizes,^34,35,36,37,38^ although measurement challenges in identifying appropriate exposure constructs make it difficult to identify both the risks and benefits of digital and social media use.^39^ Other hypotheses have included interactions between historical patterns in biological risk factors (eg, earlier maturation and puberty)^40^ and social risk factors (eg, political polarization and environmental instability limiting future outlooks).^41,42^ As new cohorts of adolescents emerge and existing cohorts transition into adulthood, it is unlikely that the value of these novel social factors will abate; thus, without investment into prevention and intervention, further acceleration of mental health problems may be expected.

### Limitations

This study is subject to limitations. Depressive symptoms were self-reported rather than ascertained using diagnostic instruments or clinical assessment. While self-reported depressive symptoms are typically reliable indicators of psychological distress, these measures have not been validated with clinical cut points for probable depression, and thus we do not know the extent to which the patterns represented here would generalize to clinical depression. We also cannot separate patterns in willingness to report depressive symptoms from true change; given that all depression diagnoses and symptoms were based on self-report (even to a clinician), there was no way to distinguish true change from willingness to report. However, the patterns observed in the MTF data were similar to those observed in other data sources, including those with diagnostic instruments^1^ and administrative records of outcomes such as suicidal self-injury,^4^ reducing the possibility that the patterns observed here were not meaningful for population mental health. Furthermore, the MTF panel has substantial attrition across follow-up waves, although statistical weights adjusted for the association of attrition with study estimates. Additionally, the baseline MTF survey is conducted only among adolescents in school, and thus we lacked data on individuals who left high school or were absent on the day of the survey. We anticipated that respondents who were not in high school likely had higher levels of depressive symptoms and other mental health problems; thus, the estimates here are likely to be conservative.

## Conclusions

The findings of this cohort study suggest that the current increase in depressive symptoms among US adolescents is swiftly shifting to an increase in young adult depressive symptoms as adolescents move into adulthood. There was significant persistence in depressive symptoms from ages 18 years to 21 to 22 years, with the recent birth cohort reporting the most stable elevated symptoms across age compared with previous birth cohorts. Given this persistence, reducing the onset of depressive symptoms through primary prevention and mental health resources during the adolescent years is critical.

## Notes

Supplement 1. **eFigure 1.** Longitudinal Prevalence of High Loneliness by Birth Cohort and Sex Among US Young Adults Born 1972 Through 2001 and Measured From 1990 Through 2019 **eFigure 2.** Longitudinal Prevalence of High Self-Derogation by Birth Cohort and Sex Among US Young Adults Born 1972 Through 2001 and Measured From 1990 Through 2019 **eFigure 3.** Longitudinal Prevalence of Low Self-Esteem by Birth Cohort and Sex Among US Young Adults Born 1972 Through 2001 and Measured From 1990 Through 2019 **eFigure 4.** Prevalence of High Loneliness at Age 19/20 and Age 21-22 Stratified by Whether the Respondents Had High Depressive Symptoms at Age 18, by Birth Cohort **eFigure 5.** Prevalence of High Self-Derogation at Age 19/20 and Age 21-22 Stratified by Whether the Respondents Had High Depressive Symptoms at Age 18, by Birth Cohort **eFigure 6.** Prevalence of Low Self-Esteem at Age 19/20 and Age 21-22 Stratified by Whether the Respondents Had High Depressive Symptoms at Age 18, by Birth Cohort **eTable 1.** Sample Size by Cohort and Wave **eTable 2.** Demographics by Birth Cohort **eTable 3.** Adjusted Odds Ratios Between Birth Cohort and Mental Well-Being at Ages 18, 19/20, and 21/22 **eTable 4.** Adjusted Odds Ratios Between Baseline Mental Health Predictors and Mental Health Outcomes at Ages 19/20 and Age 21/22 **eTable 5.** Interaction *P* values for Age 18 Predictor by Sex

Supplement 2.
Data Sharing Statement
