# Supplementary material for: Depressive Symptoms in Adolescence and Young Adulthood
Source: JAMA Netw Open. 2024 Aug 14;7(8):e2427748. doi: 10.1001/jamanetworkopen.2024.27748 (PMC11325205; doi:10.1001/jamanetworkopen.2024.27748)
Supplement: Supplement 1. — eFigure 1. Longitudinal Prevalence of High Loneliness by Birth Cohort and Sex Among US Young Adults Born 1972 Through 2001 and Measured From 1990 Through 2019 eFigure 2. Longitudinal Prevalence of High Self-Derogation by Birth Cohort and Sex Among US Young Adults Born 1972 Through 2001 and Measured From 1990 Through 2019 eFigure 3. Longitudinal Prevalence of Low Self-Esteem by Birth Cohort and Sex Among US Young Adults Born 1972 Through 2001 and Measured From 1990 Through 2019 eFigure 4. Prevalence of High Loneliness at Age 19/20 and Age 21-22 Stratified by Whether the Respondents Had High Depressive Symptoms at Age 18, by Birth Cohort eFigure 5. Prevalence of High Self-Derogation at Age 19/20 and Age 21-22 Stratified by Whether the Respondents Had High Depressive Symptoms at Age 18, by Birth Cohort eFigure 6. Prevalence of Low Self-Esteem at Age 19/20 and Age 21-22 Stratified by Whether the Respondents Had High Depressive Symptoms at Age 18, by Birth Cohort eTable 1. Sample Size by Cohort and Wave eTable 2. Demographics by Birth Cohort eTable 3. Adjusted Odds Ratios Between Birth Cohort and Mental Well-Being at Ages 18, 19/20, and 21/22 eTable 4. Adjusted Odds Ratios Between Baseline Mental Health Predictors and Mental Health Outcomes at Ages 19/20 and Age 21/22 eTable 5. Interaction P values for Age 18 Predictor by Sex [file jamanetwopen-e2427748-s001.pdf]

Supplemental Online Content

Keyes KM, Kreski NT, Patrick ME. Depressive symptoms in adolescence and young adulthood. *JAMA Netw Open*. 2024;7(8):e2427748.
doi:10.1001/jamanetworkopen.2024.27748

eFigure 1. Longitudinal Prevalence of High Loneliness by Birth Cohort and Sex Among US Young Adults Born 1972 Through 2001 and Measured From 1990 Through 2019

eFigure 2. Longitudinal Prevalence of High Self-Derogation by Birth Cohort and Sex Among US Young Adults Born 1972 Through 2001 and Measured From 1990 Through 2019

eFigure 3. Longitudinal Prevalence of Low Self-Esteem by Birth Cohort and Sex Among US Young Adults Born 1972 Through 2001 and Measured From 1990 Through 2019

eFigure 4. Prevalence of High Loneliness at Age 19/20 and Age 21-22 Stratified by Whether the Respondents Had High Depressive Symptoms at Age 18, by Birth Cohort

eFigure 5. Prevalence of High Self-Derogation at Age 19/20 and Age 21-22 Stratified by Whether the Respondents Had High Depressive Symptoms at Age 18, by Birth Cohort

eFigure 6. Prevalence of Low Self-Esteem at Age 19/20 and Age 21-22 Stratified by Whether the Respondents Had High Depressive Symptoms at Age 18, by Birth Cohort

eTable 1. Sample Size by Cohort and Wave

eTable 2. Demographics by Birth Cohort

eTable 3. Adjusted Odds Ratios Between Birth Cohort and Mental Well-Being at Ages 18, 19/20, and 21/22

eTable 4. Adjusted Odds Ratios Between Baseline Mental Health Predictors and Mental Health Outcomes at Ages 19/20 and Age 21/22

eTable 5. Interaction P-values for Age 18 Predictor by Sex

This supplemental material has been provided by the authors to give readers additional information about their work.

© 2024 Keyes KM et al. *JAMA Network Open*

eFigure 1. Longitudinal prevalence of high loneliness by birth cohort and sex among US young adults born 1972 through 2001 and measured from 1990 through 2019.

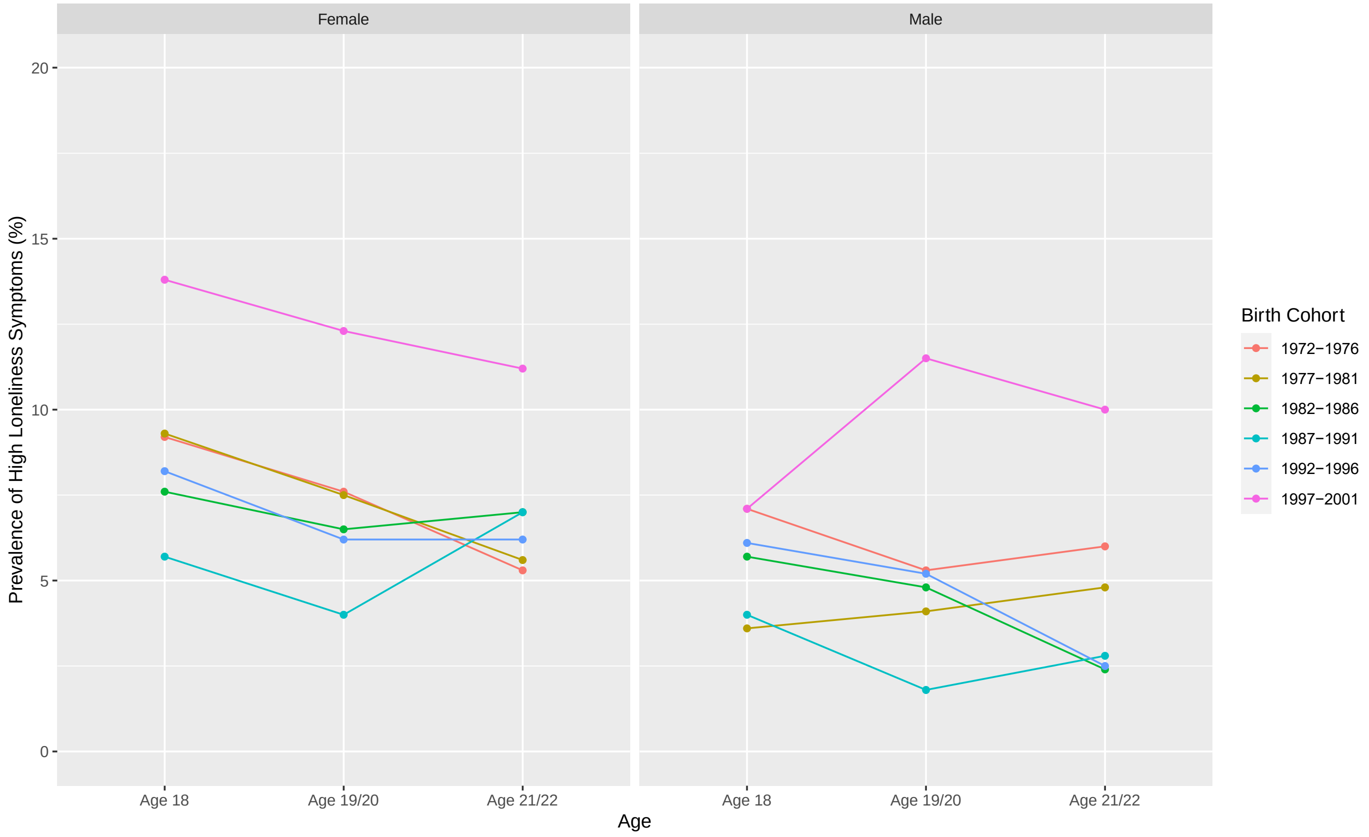

© 2024 Keyes KM et al. *JAMA Network Open*

eFigure 2. Longitudinal prevalence of high self-derogation by birth cohort and sex among US young adults born 1972 through 2001 and measured from 1990 through 2019.

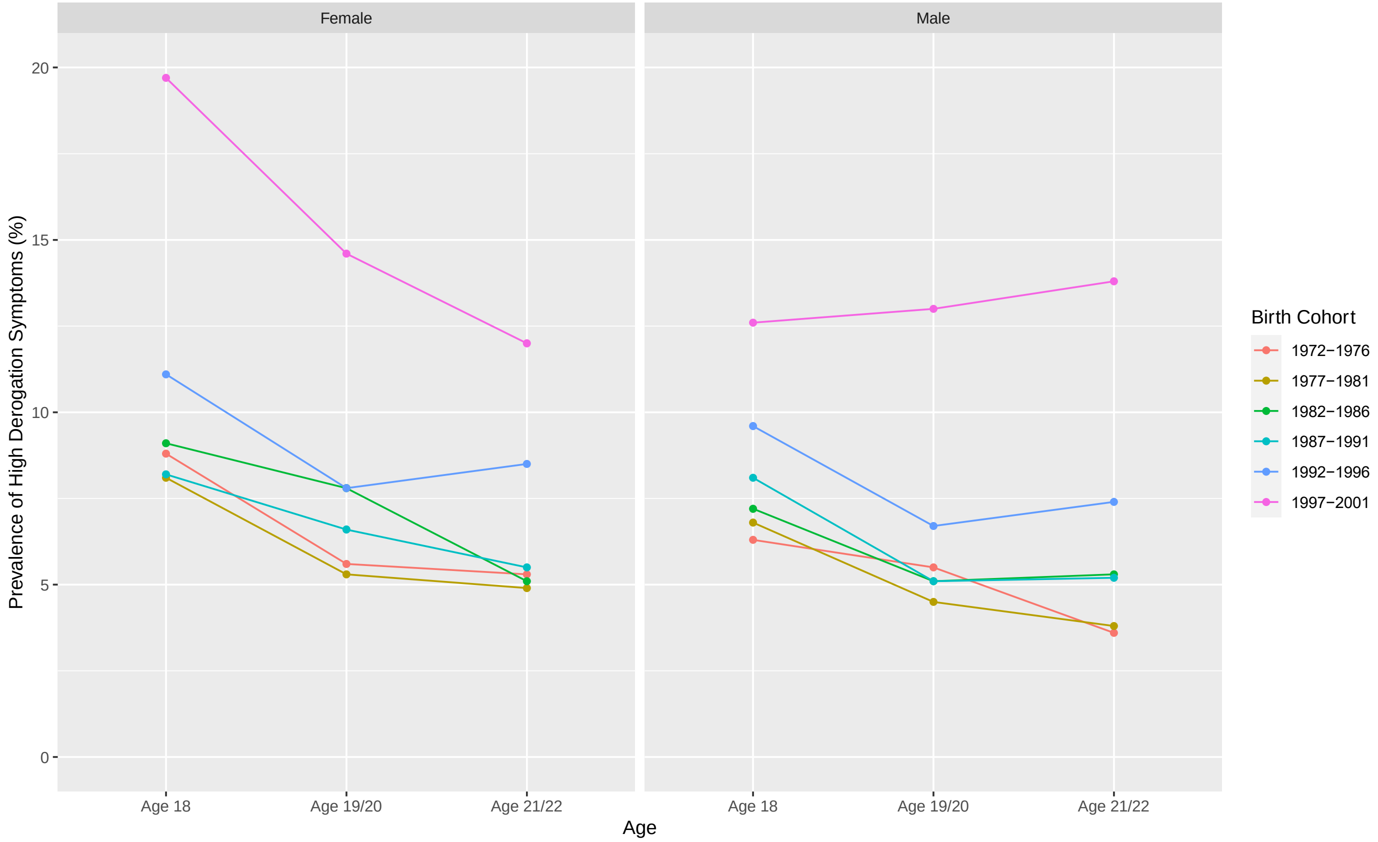

© 2024 Keyes KM et al. *JAMA Network Open*

eFigure 3. Longitudinal prevalence of low self-esteem by birth cohort and sex among US young adults born 1972 through 2001 and measured from 1990 through 2019.

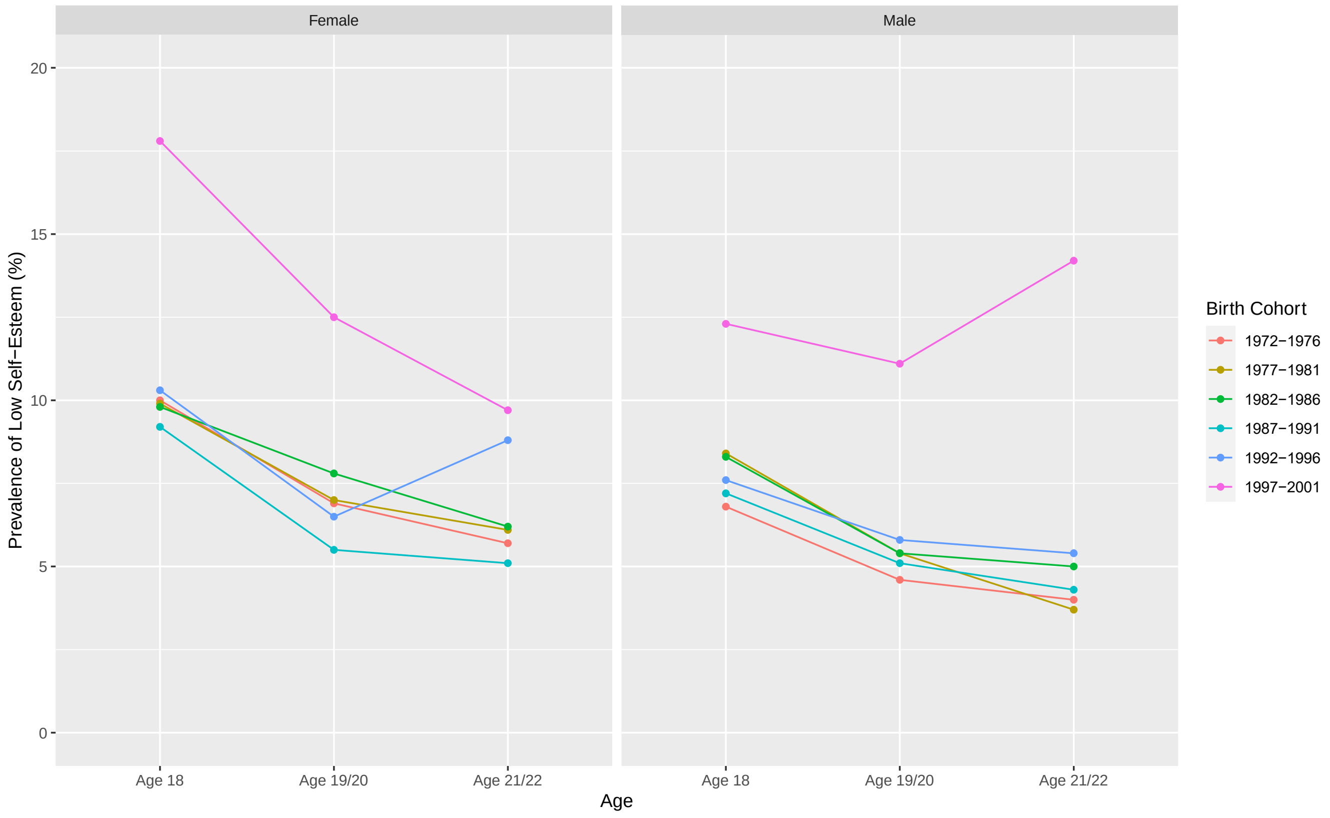

© 2024 Keyes KM et al. *JAMA Network Open*

eFigure 4. Prevalence of high loneliness at age 19/20 and age 21-22 stratified by whether the respondents had high depressive symptoms at age 18, by birth cohort.

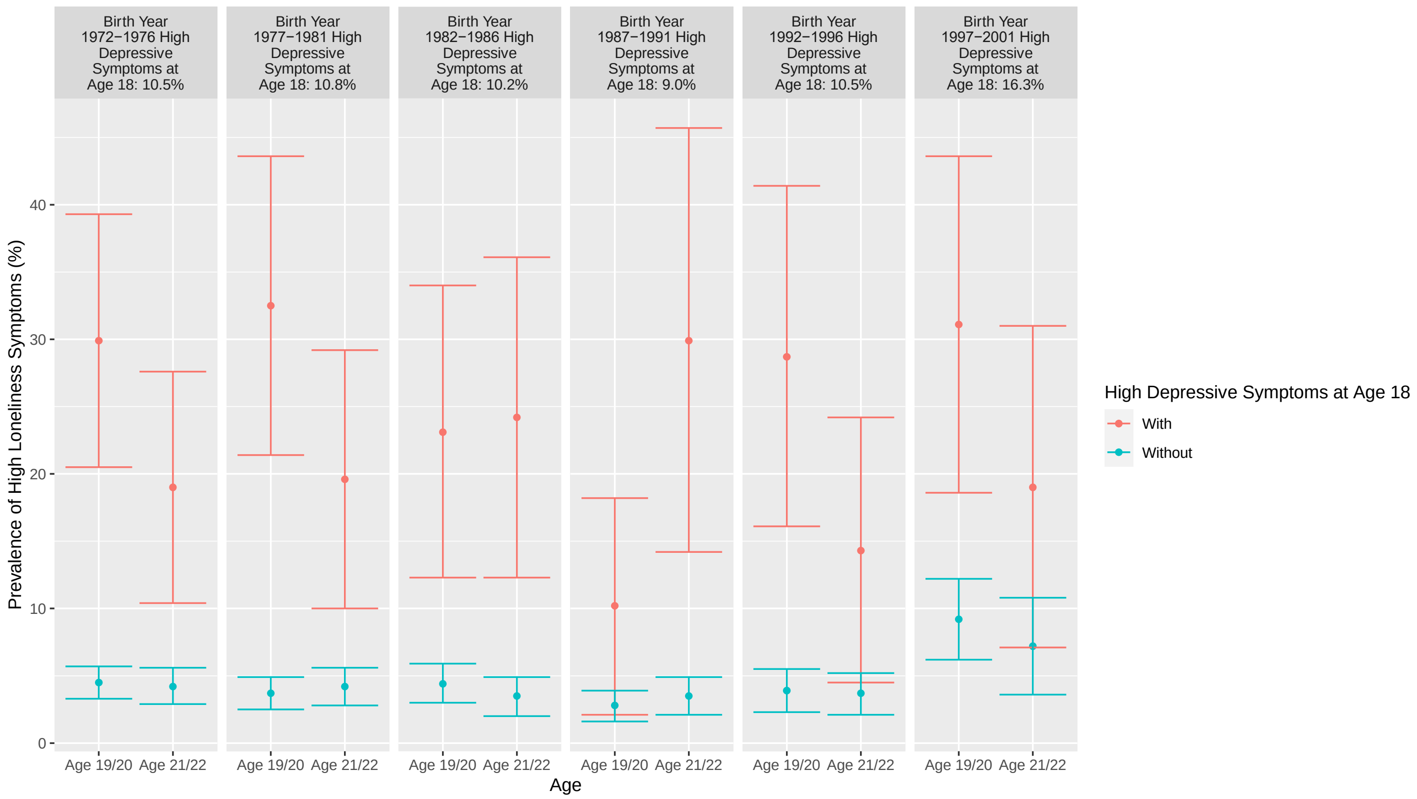

© 2024 Keyes KM et al. *JAMA Network Open*

eFigure 5. Prevalence of high self-derogation at age 19/20 and age 21-22 stratified by whether the respondents had high depressive symptoms at age 18, by birth cohort.

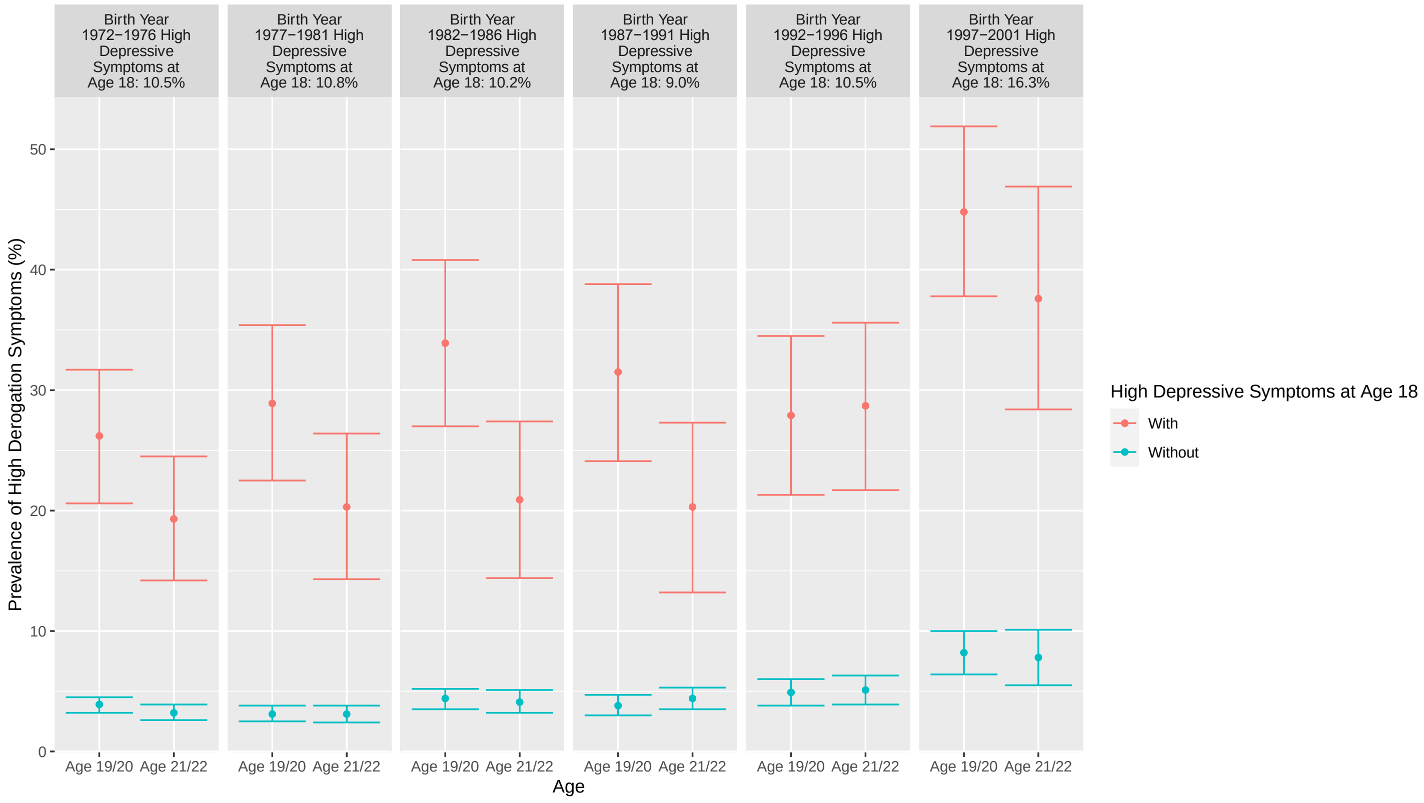

© 2024 Keyes KM et al. *JAMA Network Open*

eFigure 6. Prevalence of low self-esteem at age 19/20 and age 21-22 stratified by whether the respondents had high depressive symptoms at age 18, by birth cohort.

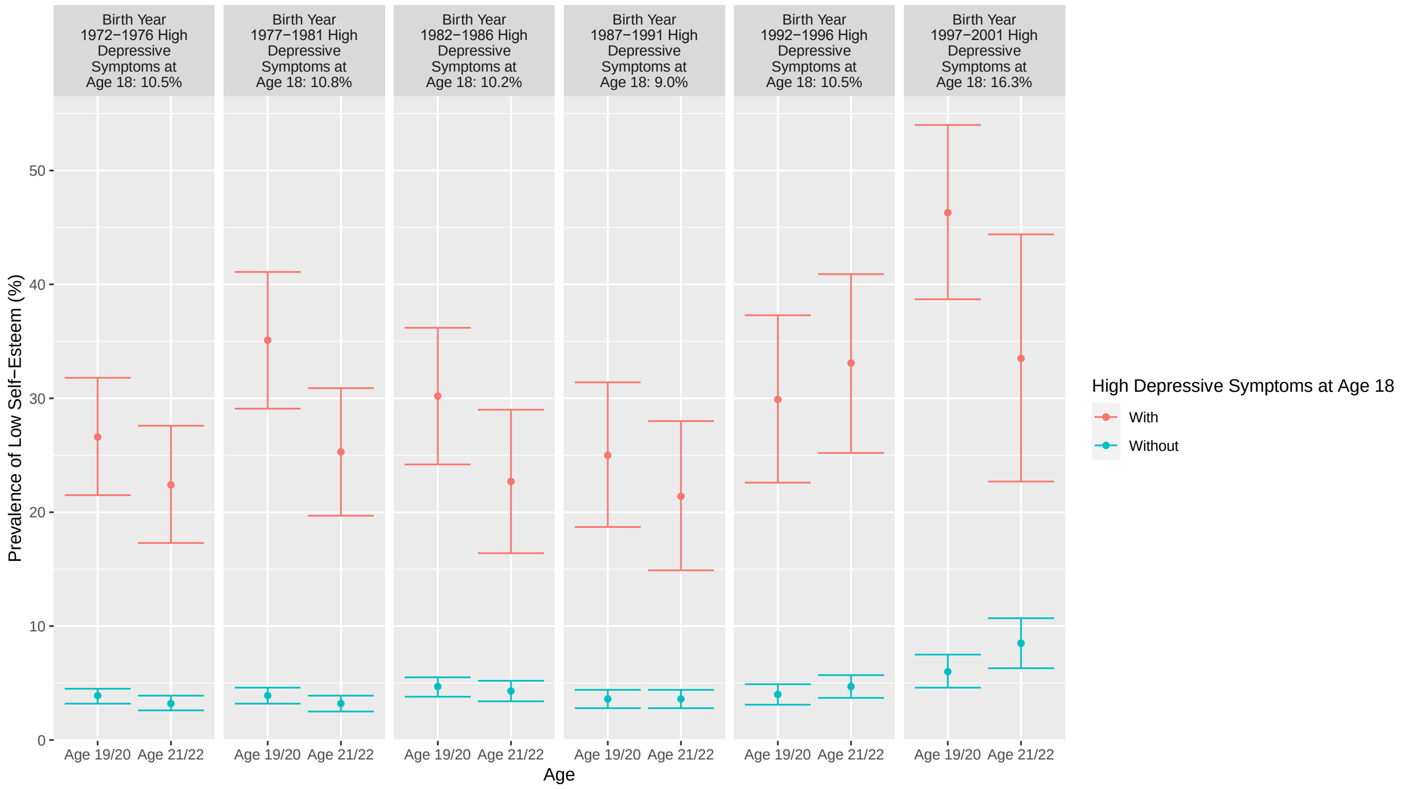

© 2024 Keyes KM et al. *JAMA Network Open*

eTable 1. Sample Size by Cohort and Wave

Birth Cohort	Baseline N	Follow-Up 1 N	Follow-Up 2 N
1972-1976	6,231	4,427	4,035
1977-1981	6,225	3,992	3,517
1982-1986	6,169	3,577	3,305
1987-1991	6,180	3,139	2,941
1992-1996	6,210	2,667	2,380
1997-2001	5,537	1,899	1,749

© 2024 Keyes KM et al. *JAMA Network Open*

eTable 2. Demographics by Birth Cohort

Demographics	Birth Cohort						
	**1972-1976**	**1977-1981**	**1982-1986**	**1987-1991**	**1992-1996**	**1997-2001**	**Overall**
**Sex**	**% (N)**	**% (N)**	**% (N)**	**% (N)**	**% (N)**	**% (N)**	**% (N)**
**Male**	50.9 (3,123)	50.0 (3,079)	48.1 (2,951)	47.9 (2,952)	49.9 (3,079)	45.3 (2,503)	48.8 (17,687)
**Female**	49.1 (3,108)	50.0 (3,146)	51.9 (3,218)	52.1 (3,228)	50.1 (3,131)	49.7 (2,766)	50.5 (18,597)
**Missing**	0 (0)	0 (0)	0 (0)	0 (0)	0 (0)	5.0 (268**)	0.7 (268**)
**Race***							
**American Indian/Alaskan Native**	1.7 (106)	1.1 (64)	1.1 (54)	1.0 (52)	1.0 (73)	1.0 (45)	1.1 (394)
**Asian and Pacific Islander**	2.1 (164)	2.6 (182)	3.2 (206)	3.8 (243)	4.2 (262)	3.9 (216)	3.3 (1,273)
**Black**	10.4 (695)	10.1 (701)	9.6 (638)	9.8 (603)	10.8 (669)	12.7 (669)	10.5 (3,975)
**Hispanic/Latino**	9.5 (552)	8.5 (556)	10.1 (632)	16.3 (1,016)	16.9 (1,111)	24.7 (1,333)	14.0 (5,200)
**Multiracial**	0 (0)	0 (0)	0 (0)	2.6 (154)	4.0 (235)	5.1 (269)	1.8 (658)
**white**	72.9 (4,473)	72.4 (4,375)	70.2 (4,275)	65.2 (4,026)	62.3 (3,822)	50.0 (2,861)	66.0 (23,832)
**Other**	2.2 (151)	3.5 (231)	3.8 (230)	0.4 (29)	0 (0)	0 (0)	1.7 (641)
**Missing**	1.2 (90)	1.8 (116)	2.1 (134)	0.8 (57)	0.7 (38)	2.6 (144)	1.5 (579)
**Parental Education**							
**1+ College Graduate Parent**	39.0 (2,500)	46.8 (2,906)	46.1 (2,968)	45.8 (2,894)	47.3 (2,979)	44.0 (2,539)	44.8 (16,786)
**0 College Graduate Parents**	53.4 (3,239)	45.3 (2,827)	45.5 (2,693)	45.0 (2,721)	42.4 (2,599)	42.2 (2,280)	45.8 (16,359)
**Missing**	7.6 (492)	7.9 (492)	8.3 (508)	9.2 (565)	10.2 (632)	13.8 (718)	9.4 (3,407)

* Until 2005, respondents selected one racial/ethnic category including “American Indian (Native American Indian)”, “Black or African-American”, “Mexican American or Chicano”, “Cuban American”, “Puerto Rican”, “Other Latin American”, “Asian American”, “White or Caucasian”, or “Other”. From 2005 onward, respondents could select multiple options from the following: “Black or African American”, “Mexican American or Chicano”, “Cuban American”, “Puerto Rican”, “Other Hispanic/Latino”, “Asian American”, “White”, “American Indian or Alaskan Native”, and “Native Hawaiian or Other Pacific Islander”. Across all years, we harmonized the categories into: American Indian/Alaskan Native, Asian/Pacific Islander, Black, Hispanic/Latino, non-Hispanic Multiracial (based on reporting multiple racial identities), and white. For respondents surveyed before 2005, we also categorized as ‘other’ those who selected that option.

**268 individuals selected “Other” or “Prefer not to answer" for gender, which were response options only available in the most recent cohorts.

© 2024 Keyes KM et al. *JAMA Network Open*

eTable 3. Adjusted* odds ratios between birth cohort and mental well-being at ages 18, 19/20, and 21/22.

	Self-derogation age 18	Self-derogation age 19/20	Self-derogation age 21/22	Low self-esteem age 18	Low self-esteem age 19/20	Low self-esteem age 21/22	Loneliness age 18	Loneliness age 19/20	Loneliness age 21/22
	OR (95% C.I.)	OR (95% C.I.)	OR (95% C.I.)	OR (95% C.I.)	OR (95% C.I.)	OR (95% C.I.)	OR (95% C.I.)	OR (95% C.I.)	OR (95% C.I.)
**All**									
**Birth cohort**									
1972-1976	REF	REF	REF	REF	REF	REF	REF	REF	REF
1977-1981	1.02 (0.86, 1.20)	0.85 (0.67, 1.07)	0.97 (0.74, 1.26)	**1.19 (1.02, 1.38)**	1.09 (0.88, 1.34)	1.09 (0.86, 1.38)	**0.74 (0.55, 1.00)**	0.85 (0.59, 1.24)	0.84 (0.55, 1.29)
1982-1986	1.07 (0.91, 1.20)	1.12 (0.89, 1.41)	1.14 (0.86, 1.50)	1.12 (0.96, 1.32)	1.21 (0.98, 1.50)	1.19 (0.92, 1.53)	0.81 (0.60, 1.08)	0.85 (0.57, 1.25)	0.84 (0.53, 1.32)
1987-1991	1.08 (0.91, 1.27)	0.94 (0.74, 1.20)	1.18 (0.91, 1.55)	1.06 (0.90, 1.24)	0.90 (0.71, 1.14)	0.93 (0.72. 1.21)	**0.54 (0.39, 0.75)**	**0.40 (0.25, 0.64)**	0.80 (0.50, 1.29)
1992-1996	**1.41 (1.20, 1.66)**	**1.31 (1.02, 1.67)**	**1.84 (1.41, 2.40)**	1.11 (0.95, 1.30)	1.13 (0.89, 1.44)	**1.56 (1.21, 2.02)**	0.81 (0.60, 1.09)	0.83 (0.54, 1.27)	0.73 (0.44, 1.22)
1997-2001	**2.45 (2.10, 2.85)**	**2.49 (1.94, 3.21)**	**3.31 (2.32, 4.70)**	**2.09 (1.80, 2.42)**	**2.15 (1.64, 2.82)**	**2.78 (1.94. 3.99)**	1.27 (0.96, 1.68)	**1.80 (1.17, 2.79)**	1.67 (0.88, 3.19)
**Boys/Men**									
**Birth cohort**									
1972-1976	REF	REF	REF	REF	REF	REF	REF	REF	REF
1977-1981	1.07 (0.83, 1.38)	0.73 (0.51, 1.05)	1.06 (0.68, 1.65)	**1.33 (1.05, 1.69)**	1.13 (0.80, 1.61)	1.01 (0.67, 1.52)	**0.43 (0.26, 0.70)**	0.54 (0.27, 1.05)	0.69 (0.35, 1.38)
1982-1986	1.15 (0.89, 1.48)	0.82 (0.57, 1.18)	**1.60 (1.03, 2.47)**	1.24 (0.97, 1.58)	1.21 (0.84, 1.74)	1.31 (0.85, 2.00)	0.65 (0.41, 1.03)	0.88 (0.47, 1.67)	0.44 (0.18, 1.07)
1987-1991	**1.32 (1.03, 1.69)**	0.85 (0.58, 1.25)	1.38 (0.90, 2.12)	1.17 (0.92, 1.49)	1.11 (0.76, 1.63)	1.02 (0.66, 1.57)	**0.50 (0.31, 0.81)**	**0.31 (0.13, 0.72)**	0.54 (0.24, 1.23)
1992-1996	**1.55 (1.21, 1.98)**	1.18 (0.81, 1.72)	**2.14 (1.39, 3.29)**	1.14 (0.89, 1.46)	1.21 (0.82, 1.80)	1.46 (0.96, 2.21)	0.76 (0.48, 1.19)	0.78 (0.39, 1.56)	**0.40 (0.17, 0.95)**
1997-2001	**2.30 (1.81, 2.91)**	**2.39 (1.60, 3.59)**	**5.03 (2.88, 8.79)**	**1.95 (1.53, 2.47)**	**2.73 (1.77, 4.22)**	**5.08 (3.00, 8.61)**	0.90 (0.58, 1.40)	1.88 (0.95, 3.71)	1.78 (0.65, 4.91)
**Girls/Women**									
**Birth cohort**									
1972-1976	REF	REF	REF	REF	REF	REF	REF	REF	REF
1977-1981	0.98 (0.78, 1.22)	0.96 (0.71, 1.29)	0.91 (0.65, 1.27)	1.08 (0.88, 1.32)	1.05 (0.81, 1.35)	1.14 (0.85, 1.53)	1.02 (0.70, 1.50)	1.03 (0.66, 1.61)	0.98 (0.56, 1.70)
1982-1986	1.01 (0.81, 1.25)	**1.41 (1.05, 1.88)**	0.85 (0.60, 1.21)	1.04 (0.84, 1.27)	1.22 (0.94, 1.59)	1.12 (0.83, 1.51)	0.92 (0.63, 1.36)	0.80 (0.49, 1.28)	1.25 (0.72, 2.18)
1987-1991	0.90 (0.72, 1.12)	1.02 (0.74, 1.39)	1.05 (0.74, 1.47)	0.96 (0.78, 1.18)	0.76 (0.56, 1.03)	0.86 (0.62, 1.21)	**0.55 (0.35, 0.86)**	**0.45 (0.25, 0.80)**	1.06 (0.59, 1.92)
1992-1996	**1.31 (1.06, 1.62)**	**1.46 (1.06, 2.00)**	**1.68 (1.20, 2.36)**	1.08 (0.88, 1.33)	1.07 (0.79, 1.46)	**1.67 (1.21, 2.30)**	0.86 (0.58, 1.28)	0.87 (0.51, 1.48)	1.10 (0.58, 2.08)
1997-2001	**2.51 (2.05, 3.07)**	**2.65 (1.92, 3.65)**	**2.27 (1.47, 3.51)**	**2.16 (1.78, 2.62)**	**1.75 (1.25, 2.44)**	1.45 (0.91, 2.31)	**1.59 (1.10, 2.30)**	1.71 (0.98, 2.97)	1.66 (0.75, 3.71)

*Adjusted for race, parental education, and pre/post-2020 indicator
Bold = $p<0.05$

© 2024 Keyes KM et al. *JAMA Network Open*

eTable 4. Adjusted* odds ratios between baseline mental health predictors and mental health outcomes at ages 19/20 and age 21/22.

Among males:

	Derogation 19/20	Derogation 21/22	Low Esteem 19/20	Low Esteem 21/22	Loneliness 19/20	Loneliness 21/22
	OR (95% C.I.)	OR (95% C.I.)	OR (95% C.I.)	OR (95% C.I.)	OR (95% C.I.)	OR (95% C.I.)
**Age 18 Depressive Symptoms**	**3.76 (2.19, 6.46)**	**3.55 (1.75, 7.22)**	**4.21 (2.41, 7.36)**	**5.24 (2.91, 9.45)**	2.82 (0.53, 15.14)	2.66 (0.03, 239.73)
**Age 18 Self-Derogation**	**7.81 (5.84, 10.44)**	**4.87 (3.44, 6.88)**	**2.48 (1.62, 3.80)**	**1.83 (1.17, 2.87)**	**2.05 (1.01, 4.18)**	1.46 (0.61, 3.48)
**Age 18 Low Self-Esteem**	**3.57 (2.38, 5.36)**	**2.66 (1.69, 4.18)**	**10.37 (7.83, 13.74)**	**6.73 (4.87, 9.29)**	**3.07 (1.48, 6.37)**	1.55 (0.76, 3.14)
**Age 18 Loneliness**	**2.21 (1.09, 4.47)**	1.92 (0.80, 4.60)	**2.40 (1.15, 5.01)**	0.75 (0.29, 1.93)	**6.93 (4.03, 11.91)**	**5.57 (2.91, 10.67)**

Among females:

	Derogation 19/20	Derogation 21/22	Low Esteem 19/20	Low Esteem 21/22	Loneliness 19/20	Loneliness 21/22
	OR (95% C.I.)	OR (95% C.I.)	OR (95% C.I.)	OR (95% C.I.)	OR (95% C.I.)	OR (95% C.I.)
**Age 18 Depressive Symptoms**	**3.09 (1.96, 4.88)**	**2.21 (1.24, 3.96)**	**2.15 (1.27, 3.64)**	**2.08 (1.13, 3.83)**	**4.82 (1.89, 12.29)**	**6.78 (1.44, 31.89)**
**Age 18 Self-Derogation**	**11.15 (8.98, 13.83)**	**8.36 (6.56, 10.64)**	**3.08 (2.31, 4.09)**	**2.84 (2.02, 3.99)**	**3.28 (2.13, 5.08)**	**3.71 (2.26, 6.11)**
**Age 18 Low Self-Esteem**	**4.22 (3.20, 5.57)**	**2.86 (2.05, 4.01)**	**10.89 (8.93, 13.28)**	**8.59 (6.87, 10.76)**	**2.91 (1.88, 4.51)**	**2.66 (1.62, 4.36)**
**Age 18 Loneliness**	**3.33 (2.13, 5.22)**	**3.37 (1.90, 5.97)**	**2.14 (1.31, 3.50)**	**2.51 (1.52, 4.15)**	**8.36 (5.78, 12.09)**	**5.18 (3.33, 8.08)**

*Adjusted for race, parental education, baseline levels of outcome, cohort and pre/post-2020 indicator
Bold = $p<0.05$

© 2024 Keyes KM et al. *JAMA Network Open*

eTable 5. Interaction p-values for age 18 predictor by sex

	Depressive	Derogation	Low Self-Esteem	Loneliness
**Depressive Symptoms Follow Up 1**	0.8043	0.9060	0.9734	0.2709
**Depressive Symptoms Follow Up 2**	0.5281	0.0647	0.2142	**<.0001**
**Self-Derogation Follow Up 1**	0.8887	**0.0333**	0.1714	0.1005
**Self-Derogation Follow Up 2**	0.8279	**0.0172**	0.2044	**0.0423**
**Low Self-Esteem Follow Up 1**	0.2539	0.3463	0.6936	0.5979
**Low Self-Esteem Follow Up 2**	0.3549	0.0636	0.2000	**0.0159**
**Loneliness Follow Up 1**	0.4321	0.1516	0.9941	0.5177
**Loneliness Follow Up 2**	0.9517	0.0662	0.2678	0.9699

Bold = p<0.05

© 2024 Keyes KM et al. *JAMA Network Open*
