# Supplementary material for: Depressive Symptoms in Adolescence and Young Adulthood
Source: JAMA Netw Open. 2024 Aug 14;7(8):e2427748. doi: 10.1001/jamanetworkopen.2024.27748 (PMC11325205; doi:10.1001/jamanetworkopen.2024.27748)
Supplement: Supplement 2. — Data Sharing Statement [file jamanetwopen-e2427748-s002.pdf]

# Data Sharing Statement

Keyes. Depressive Symptoms in Adolescence and Young Adulthood. *JAMA Netw Open*. Published August 14, 2024. doi:10.1001/jamanetworkopen.2024.27748

**Data**
**Data available:** No

**Additional Information**
**Explanation for why data not available:** Data are available through an approved IRB and data use agreement with University of Michigan
